# Reduced Plasma Bone Morphogenetic Protein 6 Levels in Sepsis and Septic Shock Patients

**DOI:** 10.3390/biomedicines12081682

**Published:** 2024-07-28

**Authors:** Niklas Schmidtner, Alexander Utrata, Patricia Mester, Stephan Schmid, Martina Müller, Vlad Pavel, Christa Buechler

**Affiliations:** Department of Internal Medicine I, Gastroenterology, Hepatology, Endocrinology, Rheumatology, and Infectious Diseases, University Hospital Regensburg, 93053 Regensburg, Germany; niklas.schmidtner@stud.uni-regensburg.de (N.S.); alexander.utrata@stud.uni-regensburg.de (A.U.); patricia.mester@klinik.uni-regensburg.de (P.M.); stephan.schmid@klinik.uni-regensburg.de (S.S.); martina.mueller-schilling@klinik.uni-regensburg.de (M.M.); vlad.pavel@klinik.uni-regensburg.de (V.P.)

**Keywords:** septic shock, SIRS, COVID-19, C-reactive protein, survival, BMP6

## Abstract

Infectious diseases are associated with low iron levels and the induction of hepcidin, the primary protein regulating cellular iron export. Bone morphogenetic protein 6 (BMP6), a key regulator of hepcidin expression, has not yet been analyzed in the plasma of patients with systemic inflammatory response syndrome (SIRS) or sepsis. An analysis of 38 SIRS, 39 sepsis, and 78 septic shock patients revealed similar levels of BMP6 in sepsis and septic shock, which were lower compared to patients with SIRS and healthy controls. Plasma BMP6 levels did not correlate with procalcitonin and C-reactive protein levels in patients with SIRS or sepsis/septic shock. Neither bacterial nor SARS-CoV-2 infections affected plasma BMP6 levels. There was no difference in BMP6 levels between ventilated and non-ventilated patients, or between patients with and without dialysis. Vasopressor therapy did not alter BMP6 levels. Survivors had plasma BMP6 levels similar to non-survivors. Due to the high variability of plasma BMP6 levels, these analyses have limited clinical relevance. Iron, ferritin, and transferrin levels were known in at least 50% of patients but did not correlate with plasma BMP6 levels. In conclusion, this study showed normal BMP6 plasma levels in SIRS, which are reduced in patients with sepsis and septic shock. This suggests that the commonly observed increase in hepcidin levels and the decline in iron levels in SIRS, sepsis, and septic shock are not due to higher BMP6.

## 1. Introduction

Sepsis is a life-threatening condition resulting from a dysregulated host response to inflammation [1,2]. Iron is a vital trace element necessary for fundamental processes in both humans and bacteria. During sepsis, iron metabolism is altered, characterized by increased iron transport and uptake into cells and decreased iron export. 

Hepcidin, a crucial regulator of iron metabolism, degrades ferroportin, the cellular iron exporter found in hepatocytes, duodenal enterocytes, and macrophages [3]. During bacterial infections, hepcidin is upregulated to limit the iron available to pathogens. Accordingly, serum iron and transferrin, an iron-transporting protein, are low in sepsis. Ferritin, which is an acute phase protein, is increased [4,5,6,7].

Hepcidin expression in hepatocytes is increased by iron, activin B, interleukin-6 (IL-6), and lipopolysaccharide (LPS) [3,7]. The upregulation of hepcidin by inflammatory stimuli is mediated by the Janus kinase (JAK)/signal transducer and the activator of the transcription 3 (STAT3) pathway in hepatocytes [8]. Iron-dependent signaling involves the binding of bone morphogenetic protein (BMP) 6 to type I and type II BMP receptors, leading to the phosphorylation of small mothers against decapentaplegic homologs (SMAD) 1/5/8. This complex then forms a complex with SMAD4, translocates to the nucleus, and activates hepcidin expression [3,7].

Patients with sepsis exhibit significantly higher plasma hepcidin levels compared to healthy controls. Elevated plasma hepcidin levels at admission were predictive of 28-day mortality in sepsis patients [9]. Tacke et al. measured serum hepcidin in patients requiring intensive care. Participants meeting the criteria for severe sepsis as defined by the American College of Chest Physicians and the Society of Critical Care Medicine Consensus Conference Committee were classified as septic, while the remaining participants were classified as non-septic [6]. This study found increased hepcidin and ferritin levels in septic patients compared to both controls and non-septic severely ill patients. Hepcidin levels positively correlated with C-reactive protein, procalcitonin, and IL-6 in both patient cohorts. Serum hepcidin levels were similar between survivors and non-survivors in this patient group [6].

Serum iron levels were also found to be reduced in patients infected with severe acute respiratory syndrome coronavirus type 2 (SARS-CoV-2) who were hospitalized in both normal wards and intensive care units [10,11]. Notably, Coronavirus Disease 2019 (COVID-19) patients had lower serum hepcidin levels compared to non-infected controls [8]. However, Zhu et al. reported increased serum hepcidin and ferritin levels in severe COVID-19 cases compared to mild cases and healthy controls [12]. In critically ill COVID-19 patients needing intensive care, high plasma hepcidin predicted mortality independently of age, inflammation, and tissue damage [13]. This association was not observed in a cohort of patients that included moderate and severe COVID-19 cases, and, here, ferritin was associated with mortality [11].

BMP6 is a master regulator of hepcidin expression [14]. Mice deficient in BMP6 exhibit low hepcidin expression and accumulate iron in their tissues [14]. 

BMP6 improves hyperglycemia and insulin resistance, and the higher expression of glucose transporter 4 as well as an increase in brown fat mass may have a role herein [15]. Recombinant BMP6 has anti-inflammatory activities, exerts anti-fibrotic effects in hepatic stellate cells, and was protective in a murine model of non-alcoholic fatty liver disease [16]. In the RAW 264.7 macrophage cell line, BMP6 was shown to induce tumor necrosis factor alpha and inducible nitric oxide expression, indicating proinflammatory effects in these immune cells [17].

BMP6 is expressed by hepatocytes, hepatic stellate cells, sinusoidal endothelial cells, and Kupffer cells [18]. Dietary iron was found to induce BMP6 expression in non-parenchymal liver cells [19] and in epithelial cells of the small intestine [20]. In microglial cells, BMP6 was induced in inflammation, with IL-6 having a central role herein [21]. BMP6 is abundant in circulation [22,23] but has, to the best of our knowledge, not been measured in patients with sepsis or septic shock. 

Our study aimed to evaluate the associations between plasma BMP6 levels and disease severity and outcomes in patients with systemic inflammatory response syndrome (SIRS), sepsis, or septic shock.

## 2. Materials and Methods

### 2.1. Study Cohort

From August 2018 to January 2024, plasma was obtained from 155 patients at the University Hospital of Regensburg. The cohort included 38 patients with systemic inflammatory response syndrome (SIRS), 39 patients with sepsis, and 78 patients with septic shock. Among this last group, 23 were infected with SARS-CoV-2, and their plasma was collected from October 2020 to January 2023. The patient categorization followed the Sepsis-3 criteria [24]. Patients who met the SIRS criteria but did not develop sepsis during their stay in the intensive care unit were classified as having SIRS [25]. Patients with multi-resistant infections, viral hepatitis, or HIV at admission to the intensive care ward were excluded. The control group was composed of 43 volunteers, including 21 males and 22 females, who were hospital employees, medical students, and relatives of the students.

### 2.2. BMP6 ELISA

Blood samples were taken 12 to 24 h after admission to the intensive care unit. EDTA was used as an anticoagulant to prepare the plasma. BMP6 levels were measured in duplicate using the human BMP6 ELISA Kit from Invitrogen (ThermoFisher Scientific, Carlsbad, CA, USA) as recommended by the manufacturer. Plasma samples were diluted 2-fold. Mean values were used for calculations.

### 2.3. Statistical Analysis

Boxplots display the minimum, maximum, first quartile, third quartile, and median. Outliers are depicted as circles or asterisks. Tables list the median, minimum, and maximum values. The data were analyzed using the Mann–Whitney U-test, Chi-Square test, Kruskal–Wallis test, and Spearman’s correlation with the IBM SPSS Statistics 26.0 program. A *p*-value of less than 0.05 was considered significant.

## 3. Results

### 3.1. BMP6 in Plasma of Controls and of SIRS/Sepsis Patients 

ELISA was used to measure plasma BMP6 levels in 38 patients with SIRS and 117 patients with sepsis or septic shock (Table 1). The control cohort was of similar age but included more women than the sepsis/septic shock cohort. Patients with sepsis or septic shock had a higher body mass index (BMI) and a greater number of immature granulocytes, with no differences in other listed laboratory values (Table 1).

**Figure 1:**
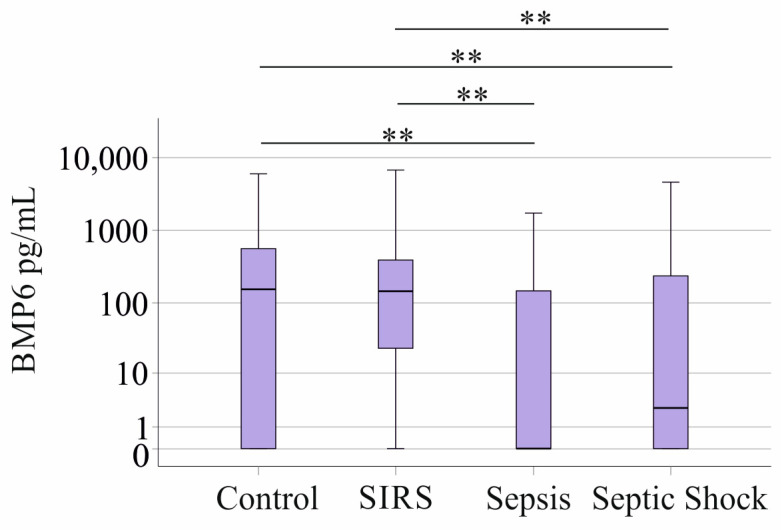
BMP6 in plasma of controls, patients with SIRS, patients with sepsis, and patients with septic shock. ** *p* < 0.01.

Plasma BMP6 concentrations were 145 (0–6746) pg/mL in the 38 patients with SIRS, 0 (0–1728) pg/mL in the 39 sepsis patients, and 6 (0–4600) pg/mL in the 78 patients with septic shock. BMP6 levels of the 43 controls were 154 (0–5974) pg/mL and were similar to the levels in SIRS patients. BMP6 in the plasma of sepsis and septic shock patients was decreased compared to SIRS patients and healthy controls (Figure 1). Because of this difference between SIRS and sepsis/septic shock, further calculations of BMP6 levels were performed separately in these cohorts.

Both men and women in the SIRS (26 males and 12 females) and the sepsis/septic shock group (84 males and 33 females) had comparable BMP6 levels (*p* = 0.593 and *p* = 0.138, respectively). Male controls exhibited higher plasma BMP6 in comparison to female controls (*p* = 0.031).

There was no correlation between BMP6 plasma levels and age in the controls and the SIRS cohorts. The Spearman correlation coefficient was r = −0.096 (*p* = 0.542) for controls and r = −0.076 (*p* = 0.652) for patients with SIRS. A negative correlation was observed for the sepsis/septic shock group (r = −0.190, *p* = 0.040). BMP6 levels of the patients were not related to the BMI (r = 0.104, *p* = 0.541 for SIRS and r = −0.152, *p* = 0.101 for sepsis/septic shock).

### 3.2. Ferritin, Transferrin, and Iron of Patients with SIRS, Sepsis, and Septic Shock

Blood iron, ferritin, and transferrin were measured in 77, 92, and 81 patients, respectively. The cohort included 28 patients with liver cirrhosis, who often have high ferritin and low transferrin levels [26]. In the subcohort where iron, ferritin, and transferrin levels were known, the 23 patients with cirrhosis tended to have higher ferritin (*p* = 0.084), significantly higher iron (*p* = 0.014) and lower transferrin levels (*p* = 0.002). BMP6 levels of these two groups were similar (*p* = 0.260). For further analysis of iron, ferritin, and transferrin, patients with liver cirrhosis were excluded. 

The reference value for ferritin and transferrin is <350 U/L. Ferritin was 963 (41–22,787) U/L, and transferrin was 120 (55–266) U/L in the SIRS/sepsis/septic shock patients. The range for normal iron is 33–192 µg/dL and was 42 (12–311) µg/dL in the SIRS/sepsis/septic shock patients, indicating that median values were close to the lower end of the normal range. 

Ferritin (*p* = 0.561), iron (*p* = 0.273), and transferrin (*p* = 0.146) were similar in patients with SIRS, sepsis, and septic shock. BMP6 was still lower in SIRS (*p* = 0.006) compared to sepsis/septic shock when patients with liver cirrhosis were excluded.

BMP6 did not correlate with ferritin (r = −0.024, *p* = 0.823), iron (r =−0.084, *p* = 0.467), or transferrin (r = −0.006, *p* = 0.958) in SIRS/sepsis/septic shock patients when patients with liver cirrhosis were excluded. This did not change when analysis was performed in the sepsis/septic shock subgroup. 

### 3.3. Correlations with Inflammation Markers and Leukocytes 

BMP6 did not correlate with leukocyte numbers, procalcitonin, CRP, or IL-6 in the sepsis/septic shock group (*p* > 0.05 for all) or in the SIRS/sepsis/septic shock cohort when patients with liver cirrhosis were excluded (Table 2). In this cohort, iron negatively correlated with leukocyte count, neutrophils, and monocytes, and transferrin positively correlated with lymphocyte number (Table 2).

### 3.4. BMP6 in Plasma of Patients Stratified for Underlying Diseases and Infectious Diseases

**Figure 2:**
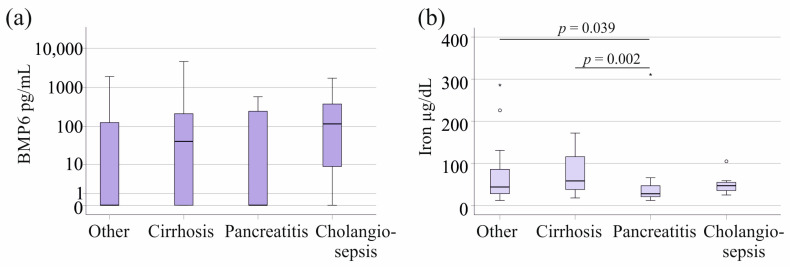
BMP6 and iron in plasma of patients with sepsis/septic shock stratified for underlying diseases and causes of sepsis/septic shock. (**a**) Plasma BMP6 levels of sepsis/septic shock patients with liver cirrhosis, pancreatitis, or cholangiosepsis. (**b**) Serum iron levels of SIRS/sepsis/septic shock patients with liver cirrhosis, pancreatitis, or cholangiosepsis. Outliers are depicted as circles (mild outliers) or asterisks (extreme outliers).

In the sepsis/septic shock cohort, twenty-four patients had pancreatitis and seven had cholangitis. There was no significant difference in plasma BMP6 levels across these patient groups (*p* = 0.425, Figure 2a). In the SIRS cohort, nine patients had pancreatitis and three had cholangitis. There was no significant difference in plasma BMP6 levels across these patient groups as well (*p* = 0.983). A total of 19 septic patients and nine patients with SIRS suffered from liver cirrhosis; however, this pathology did not affect plasma BMP6 levels compared to the plasma levels of patients without cirrhosis (Figure 2a).

Associations of ferritin and transferrin with pancreatitis and cholangitis were not observed in SIRS/sepsis/septic shock patients. Iron was low in pancreatitis in comparison to patients with other underlying diseases (*p* = 0.039) (Figure 2b). Iron was high in cirrhosis, and this was significant in comparison to patients with pancreatitis (Figure 2b).

Infections that led to sepsis or septic shock were pulmonary (forty-nine patients) and urinary tract infections (nine patients) in the sepsis/septic shock cohort. In the SIRS cohort, four patients had pulmonary infections and six had urinary tract infections. Plasma BMP6 levels were similar between these groups (*p* = 0.665 for sepsis/septic shock and *p* = 0.935 for SIRS).

Associations of ferritin, iron, and transferrin with pneumonia and urosepsis were not observed in SIRS/sepsis/septic shock patients (*p* > 0.05 for all).

**Figure 3:**
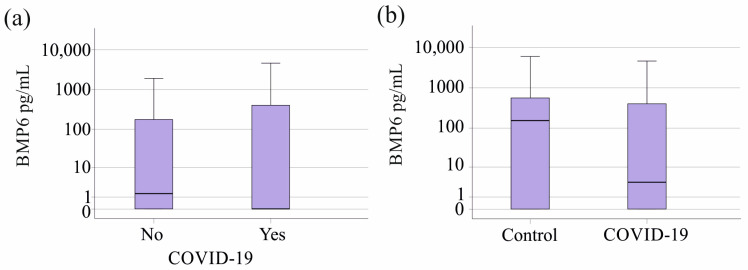
BMP6 in plasma of controls as well as sepsis/septic shock patients stratified for SARS-CoV-2. (**a**) Plasma BMP6 levels of the 23 patients with SARS-CoV-2 infection in contrast to patients not infected by this virus. (**b**) Plasma BMP6 levels of the 23 patients with SARS-CoV-2 infection in contrast to the 43 healthy controls.

The 23 COVID-19 patients exhibited plasma BMP6 levels comparable to the sepsis/septic shock patients without a SARS-CoV-2 infection (*p* = 0.250, Figure 3a). Plasma BMP6 levels of COVID-19 patients tended to be lower in comparison to the healthy controls (*p* = 0.097, Figure 3b). Ferritin, iron, and transferrin of patients with and without COVID-19 were similar (*p* > 0.05 for all).

### 3.5. Plasma BMP6 in Relation to Vasopressor Therapy and Therapeutic Interventions

Furthermore, the associations between plasma BMP6 levels and the need for dialysis, ventilation, or vasopressor treatment in patients with sepsis/septic shock were examined. Patients requiring dialysis had similar plasma BMP6 levels compared to those not on dialysis. Plasma BMP6 concentrations of patients with the need for ventilation or vasopressor therapy tended to be increased (Table 3). In the SIRS cohort, very few patients needed interventions or vasopressor therapy (Table 3), making statistical tests less meaningful.

Dialysis was related to a trend for higher ferritin (*p* = 0.087, 31 patients) and lower transferrin (*p* = 0.085, 28 patients) levels. Associations of ferritin, transferrin, and iron with ventilation and vasopressor therapy were not observed. 

### 3.6. Plasma BMP6 Levels in Gram-Negative and Gram-Positive Infection

**Figure 4:**
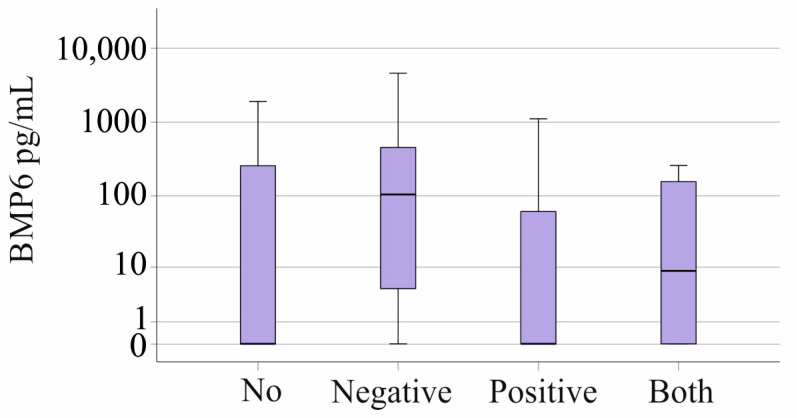
BMP6 in plasma of patients with sepsis/septic shock stratified for type of bacterial infection.

Plasma BMP6 levels in sepsis/septic shock patients without bacterial infection were comparable to those with Gram-negative (twelve patients), Gram-positive (nineteen patients), or mixed bacterial infections (four patients) (*p* = 0.250; Figure 4). There was no significant difference between the twelve Gram-negative, the six Gram-positive, and the non-infected patients with SIRS (*p* = 0.193). Ferritin, iron, and transferrin levels were not related to bacterial infection in patients with SIRS/sepsis/septic shock (*p* > 0.05 for all).

### 3.7. Plasma BMP6 Levels and Survival

**Figure 5:**
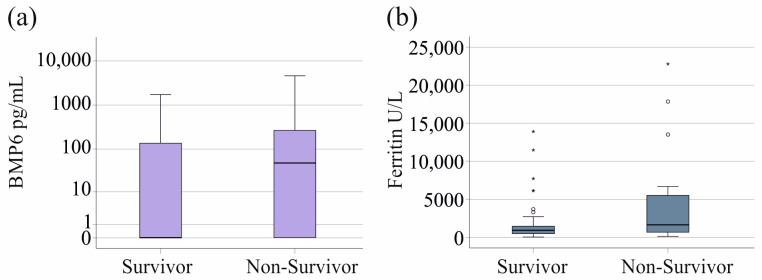
BMP6 and ferritin in blood of survivors and non-survivors in patients with sepsis/septic shock. (**a**) BMP6 plasma of survivors and non-survivors in patients with sepsis/septic shock. (**b**) Ferritin of survivors and non-survivors in patients with SIRS/sepsis/septic shock, excluding patients with liver cirrhosis. Outliers are depicted as circles (mild outliers) or asterisks (extreme outliers).

In the sepsis/septic shock cohort, 38 patients did not survive. Surviving and non-surviving patients had similar plasma BMP6 levels (*p* = 0.130, Figure 5a). All patients with SIRS survived. The 20 patients with known ferritin levels who died had increased ferritin levels (*p* = 0.060, Figure 5b).

## 4. Discussion

To our knowledge, this is the first study to analyze plasma BMP6 levels in critically ill patients. The current analysis showed that plasma BMP6 levels are normal in SIRS patients and are reduced in patients with sepsis and septic shock. BMP6 does not appear to contribute to lower iron levels in critically ill patients [6,7].

Iron and transferrin levels of the patients were lower, and ferritin levels were higher in SIRS/sepsis/septic shock patients when compared to the normal ranges of these measures. This is in accordance with previous studies [4,5,6,7,26]

BMP6 is the master regulator of hepcidin [14]. Hepcidin was elevated during sepsis and was positively associated with the degree of inflammation and the number of SIRS criteria [27]. Serum hepcidin levels were higher in severely ill septic patients compared to non-septic patients, and both groups of patients had elevated hepcidin levels compared to healthy controls [6]. Our data show that plasma BMP6 levels in sepsis and septic shock patients are reduced.

LDN-193189 and oversulfated heparins inhibit hepcidin expression by interacting with the BMP6-SMAD pathway. Both inhibitors reduced hepcidin levels in uninfected mice; however, they were not effective in mice systemically infected with either *Escherichia coli* or *Salmonella typhimurium* [28]. The authors of this study suggested that other pathways compensate for the dysfunctional BMP6 signaling in vivo [28]. This animal experiment [28] and our study in critically ill patients, therefore, suggest that the BMP6 signaling pathway may have a limited role in regulating hepcidin levels in severe inflammatory disease. Accordingly, there is experimental evidence that mice lacking BMP6 were still able to induce hepcidin production when challenged with LPS [29], and that STAT3 signaling in hepatocytes was required for the induction of hepcidin by inflammatory stimuli [30].

Given that BMP6 is not related to hepcidin and iron metabolism in severe illness, it is not unexpected that BMP6 plasma levels of patients with sepsis/septic shock were not correlated with CRP, procalcitonin, or white blood cell counts. Recombinant BMP6 has been shown to have anti-inflammatory as well as proinflammatory properties [16,17]. However, associations with CRP were not observed in septic patients. Iron, ferritin, and transferrin did not correlate with laboratory measures of inflammation. Iron negatively correlated with neutrophils, which is in accordance with low iron and a higher number of these innate immune cells in sepsis [6,31]. Whether the negative association of iron and monocyte count is related to the essential role of these cells for iron uptake and recycling requires further study [32]. Transferrin and lymphocyte numbers decrease in sepsis [3,33] and were positively correlated in the patients with severe illness, which is principally in line with the role of transferrin for lymphopoiesis [34]. 

BMP6, iron, and transferrin levels of survivors and non-survivors were similar. Ferritin tended to be induced in patients who died, and this has been described previously [6,9].

Patients with SIRS had normal plasma BMP6 levels. The pathways contributing to lower levels in patients with sepsis/septic shock have not been defined so far. Iron has been described as an inducer of BMP6 expression in the liver [35]. Anemia resulting from inflammatory processes has a high prevalence in critically ill patients [36]; however, iron levels in SIRS and sepsis/septic shock patients were similar, indicating that iron does not contribute to the decline in BMP6 in sepsis/septic shock. 

High variability in plasma BMP6 levels was observed in controls and patients, and this has also been noted in other studies that have examined different control and patient groups [22,23]. Although BMP6 was lower in sepsis and septic shock compared to SIRS, it is not a suitable biomarker for the diagnosis of sepsis due to the high variability of plasma BMP6 levels.

Plasma BMP6 levels in sepsis/septic shock patients with positive blood cultures were not altered, and this excludes BMP6 as a marker of bacterial infection. Furthermore, underlying conditions that lead to SIRS or sepsis, such as cholangitis, pneumonia, or urinary tract infections, did not affect plasma BMP6 levels. This suggests that lower plasma BMP6 levels are associated with sepsis itself rather than the triggering condition. Notably, patients with pancreatitis had low iron levels, and a role of the pancreas in iron homeostasis has been described [37]. Again, BMP6 levels did not change in pancreatitis. Likewise, liver cirrhosis did not affect plasma BMP6 levels. Arndt et al. analyzed BMP6 mRNA and protein levels in the human cirrhotic liver and showed the normal expression of BMP6 [16]. Hepatoprotective effects of BMP6 have been described in non-alcoholic fatty liver disease [16], indicating an association of BMP6 with metabolism. BMP6 improves hyperglycemia and insulin resistance [15], and this may ameliorate the severity of metabolic liver disease. Critically ill patients often have hyperglycemia because of impaired insulin activity [38]; however, whether low BMP6 levels of patients with sepsis/septic shock contribute to metabolic pathophysiology in these patients requires further study. Patients with liver cirrhosis tended to have higher ferritin, to have significantly higher iron and reduced transferrin levels [26]. BMP6 levels did not change with cirrhosis, excluding the role of BMP6 for the altered levels of these iron parameters in cirrhosis.

SARS-CoV-2 infection can lead to sepsis [39,40], most of the COVID-19 patients had septic shock. COVID-19 patients showed a trend towards reduced BMP6 levels when compared to healthy controls, suggesting that BMP6 is low due to the severe illness of SARS-CoV-2 infected patients, and, accordingly, plasma BMP6 levels of sepsis/septic shock patients with and without COVID-19 did not differ. Again, the wide variation in plasma BMP6 levels between controls and patients may have prevented this effect from being significant. Iron, ferritin, and transferrin levels of patients with and without COVID-19 did not differ, showing that disturbed iron homeostasis is related to critical illness rather than a specific effect of SARS-CoV-2 infection. 

Patients on dialysis did not have altered BMP6 levels compared to patients without dialysis. These patients tended to have higher ferritin and lower transferrin levels; however, this effect was not significant. There was a trend towards higher plasma BMP6 levels in patients requiring mechanical ventilation or vasopressor therapy, whereas measures of iron homeostasis did not change. 

Plasma BMP6 in the patients did not correlate with BMI or differ between sexes. There was a modest negative correlation of plasma BMP6 with age in the sepsis/septic shock cohort, which was not seen in SIRS and controls. The latter two cohorts were small, which may have prevented the identification of these associations. 

Male controls had higher plasma BMP6 levels compared to healthy females. This sex difference was not observed in the patient cohort, suggesting that the modest effect of sex on plasma BMP6 levels disappears in critical illness.

Iron deficiency impairs hemoglobin synthesis and immune function, both of which are critical in septic patients [41]. Anemia is a common finding in septic patients, and blood transfusion has been found to improve outcomes [33]. Evidence from experimental studies [28,29] and our finding of low BMP6 in sepsis/septic shock patients indicate that blocking BMP6 pathways may not be an effective option for treating anemia in septic patients.

A major limitation of this study was the small number of patients with SARS-CoV-2 infection and the small subgroup of patients without the need for dialysis, mechanical ventilation, or vasopressor therapy. Due to the high variability of plasma BMP-6 levels, these analyses are of limited clinical significance. We also analyzed common measures of iron metabolism in relation to these characteristics, and, for this reason, BMP6 data are also provided. Plasma hepcidin levels were not measured in the present study. Hepcidin increases in parallel with ferritin in healthy women [42] and is affected by liver cirrhosis and inflammation; it currently does not provide further diagnostic value over ferritin, iron, and transferrin levels [43].

## 5. Conclusions 

The current study shows that sepsis and septic shock patients have reduced plasma BMP6 levels, while levels are normal in SIRS patients. Although BMP6 is not suitable as a biomarker due to large variations in plasma, our observation has pathophysiological relevance.

## Figures and Tables

**Table 1:** This Table presents the characteristics of systemic inflammatory response syndrome (SIRS) patients and the patients with sepsis or septic shock. Median values, along with minimum and maximum values in parentheses, are provided. Statistical significance is denoted as follows: * *p* < 0.05, *** *p* < 0.001 for comparison of the SIRS and sepsis/septic shock patients, and ^&&^ *p* < 0.01 for comparison of controls and sepsis/septic shock patients.

Parameters	SIRS	Sepsis/Septic Shock	Controls
Males/Females	26/12	84/33 ^&&^	21/22 ^&&^
Age (years)	59 (29–88)	60 (21–93)	56 (21–86)
Body Mass Index (kg/m^2^)	24.5 (18.3–51.4) *	27.5 (15.4–55.6) *	not defined
SIRS/Sepsis/Septic Shock	38/0	0/39/78	not defined
C-reactive protein mg/L	155 (12–486)	162 (18–697)	not defined
Procalcitonin ng/mL	1.44 (0.05–270.00)	1.14 (0.06–114.40)	not defined
Leukocytes n × 10^9^/L	10.33 (0.06–37.38)	10.30 (0.28–1586.00)	not defined
Neutrophils n/nL	6.33 (1.46–29.73)	8.20 (0–70.20)	not defined
Basophils n/nL	0.04 (0–0.38)	0.04 (0–0.90)	not defined
Eosinophils n/nL	0.14 (0–2.89)	0.10 (0–8.80)	not defined
Monocytes n/nL	0.66 (0.02–3.59)	0.79 (0.0–45.00)	not defined
Lymphocytes n/nL	0.87 (0.10–2.79)	0.96 (0.08–28.60)	not defined
Immature Granulocytes n/nL	0.04 (0.01–2.00) ***	0.19 (0.0–6.19) ***	not defined

**Table 2:** Correlation coefficient (r) and *p*-values for plasma BMP6, ferritin, iron, and transferrin levels, and their associations with leukocyte numbers and clinical markers of inflammation in SIRS/sepsis/septic shock patients without liver cirrhosis. Statistical test used: Spearman correlation.

Biomarker	BMP6		Ferritin		Iron		Transferrin	
	r	*p*-Value	r	*p*-Value	r	*p*-Value	r	*p*-Value
Leukocytes	−0.003	0.975	−0.131	0.214	−0.331	0.003	0.133	0.236
Neutrophils	−0.082	0.374	−0.011	0.915	−0.292	0.011	0.054	0.635
Basophils	0.070	0.446	−0.011	0.921	−0.067	0.565	0.062	0.586
Eosinophils	−0.049	0.590	−0.026	0.805	−0.027	0.819	−0.082	0.468
Monocytes	0.002	0.985	−0.044	0.680	−0.236	0.040	0.147	0.192
Lymphocytes	−0.026	0.776	−0.073	0.493	0.062	0.595	0.329	0.003
Immature Granulocytes	−0.039	0.672	0.122	0.252	−0.093	0.429	0.077	0.499
Procalcitonin	0.026	0.779	0.026	0.810	−0.003	0.982	−0.198	0.083
C-reactive protein	0.027	0.762	0.138	0.189	−0.214	0.062	−0.093	0.411
IL-6	0.155	0.090	−0.096	0.372	−0.130	0.269	−0.270	0.017

**Table 3:** Plasma BMP6 levels of sepsis/septic shock and SIRS patients with/without dialysis, ventilation and vasopressor therapy. The number of patients treated is given in “N”, the percent relative to the whole cohort in brackets, and the respective *p*-values are listed.

Intervention/Drug	Sepsis/Septic Shock Patients		SIRS Patients	
	N	*p*-Value	N	*p*-Value
Dialysis	52 (44%)	0.411	2 (5%)	-
Ventilation	91 (78%)	0.089	4 (11%)	0.600
Vasopressor therapy	92 (79%)	0.102	4 (11%)	0.158

## Data Availability

Data supporting reported results can be obtained from the corresponding author.
